# Supplementary material for: Competencies in the Robotics of Care for Nursing Robotics: A Scoping Review
Source: Healthcare (Basel). 2024 Mar 8;12(6):617. doi: 10.3390/healthcare12060617 (PMC10970049; doi:10.3390/healthcare12060617)
Supplement: Supplementary file 1 [file healthcare-12-00617-s001.zip › HC ROB Supplementary Table S1.docx]

**Supplementary Table S1.** Table of analysis of selected articles.

| **ID (PubMed)** | **Bibliographic information (authors, year, country)** | **Article type** | **Aim of the investigation** | **Thematic classification** | | | | |
| --- | --- | --- | --- | --- | --- | --- | --- | --- |
|  |  |  |  | **Educational field** | | **Clinical field** | | **Theoretical, leadership, future** |
|  |  |  |  | **Undergraduate** | **Graduate and Postgraduate** | **Hospital field** | **Primary Health field** |  |
| 28544354 | Salzmann-Erikson & Eriksson (2017) Sweden  [27] | Letter to the Editor | The need to involve nursing in the management of the care robot process in elderly care. |  | Competencies in geriatric nursing. New infrastructure and education to address these new demands. | Focuses on geriatric care | |  |
| 29857375 | Strudwick, Hubert & Gehrs (2018) Canada  [20] | Overview | Provide an opportunity for a future nurse leader to develop nursing informatics competencies |  | Recquired graduate level | Hospital |  | Nursing leaders' informatics competencies |
| 31149911 | Monsen et al. (2019) United States of America  [21] | Feature article | Report the results of a study examining the alignment of nursing informatics education and health informatics education | Graduate-level certification | Master's degree |  |  |  |
| 34920498 | Lozada-Perezmitre et al. (2021) Mexico  [32] | Descriptive study | Validation of the Self-Assessment of Nursing Informatics Competencies Scale (SANICS) |  |  |  |  |  |
| 29309336 | Dunn Lopez et al. (2018) United States of America  [30] | Section editor | Highlight a sampling about a published work of Health Systems, Policy and Informatics (HSPI) RIG |  |  |  |  | Nurse researches have a timely opportunity to design tools that leverage data to create decisions aids of evidence-based nursing practice |
| 29314612 | Liu & Aungsuroch (2017) Thailand  [35] | Systematic search | Analyze the English full-text peer-reviewed published articles from the past 10 years | Registered nurses competency in the global community |  |  |  | Informatics as one component of nursing competency |
| 30639937 | Kim (2019) United States of America  [28] | Contemporary issues | Discuss a conceptual framework for an interdisciplinary education in engineering and nursing health informatics | Students graduate |  |  |  | Instructor profile: 1. An engineer who has completed a post-doctoral training in healthcare; 2. a nurse who has earned a dual graduate degree in nursing and biomedical engineering. "Learn by doing". |
| 34315050 | Kaihlanen et al. (2021) Finland  [36] | A cross-sectional study | Examining an increase in nursing informatics competencies following an educational initiative | Registered nurses |  |  |  | Nursing informatics competence (four items): 1) terminology-based documentation; 2) patient-related digital work; 3) general IT competency; 4) electronic documentation according to structured national headings |
| 34920525 | Jing et al. (2021) China  [31] | Descriptive correlational research design |  |  | Majority of participants minimum bachelor's degree | Public hospital (5) |  | Theoretical framework because of leadership: transformational leadership; nursing informatics competency and innovation self-efficacy |
| 29857387 | Condor, Sánchez Álvarez & Bidman (2018) Peru  [37] | Search within the websites of all the universities |  | Undergraduates nurses |  |  |  | The competency in nursing informatics should improve |
| 34920491 | Choi, Bove & Tarte (2021) United States of America  [33] | Descriptive study |  |  |  |  | Graduate students in family nurse practitioner |  |
| 34920501 | Chen et al. (2021) China  [38] | Literature research and nominal group technique |  | Experts involving nursing management, nursing education, clinical nursing and medical informatics |  |  |  |  |
| 35485946 | Brian Galacio (2022) United States of America  [34] | Pretest-posttest, quasi-experimental design was used for the study. | Analyze the informatics competencies of nurse practitioners, including nurses training to become nurse practitioners, before and after completing an online learning module in nursing informatics. | NPs and NP students | |  |  |  |
| 35673238 | Hübner et al. (2022) Germany, Finland and Portugal  [29] | eHealth4all@eu to present the pipeline showcasing its stages and to exemplify how to develop a course in clinical data analytics. |  |  | Master and PhD students |  |  |  |
| 36356324 | Reid et al. (2022) Australia  [39] | A scoping review protocol | This scoping review aims to review contemporary published literature on Nursing Informatics education in undergraduate nursing education. | Undergraduate nursing education |  |  |  |  |
| 28106601 | Borycki et al. (2017) Canada  [40] | Review literature | Extends nursing informatics competencies to include those focused on the area of technology-induced errors and HIT safety. | Undergraduate level and extend to the graduate level to include masters and doctorally prepared nurses in informatics | |  |  |  |
