# Supplementary material for: Competencies in the Robotics of Care for Nursing Robotics: A Scoping Review
Source: Healthcare (Basel). 2024 Mar 8;12(6):617. doi: 10.3390/healthcare12060617 (PMC10970049; doi:10.3390/healthcare12060617)
Supplement: Supplementary file 1 [file healthcare-12-00617-s001.zip › HC ROB Supplementary Table S3.docx]

**Supplementary Table S3.** Examples of competencies and programme outcomes in relation with robotics competencies.

| **ID** | **References for competencies and learning outcomes** |
| --- | --- |
|  | **Examples of competencies and programme outcomes [16,21,22,23,24]** |
| 1 | Develops new framework(s) for use in informatics.[16]  Ability to develop, to design new and complex products (devices, artefacts, etc.), processes and systems, with specifications incompletely defined and/or competing, that require integration of knowledge from different fields and non-technical – societal, health and safety, environmental, economic and industrial commercial – constraints; to select and apply the most appropriate and relevant design methodologies or to use creativity to develop new and original design methodologies.[22]  Possess and understand the knowledge that makes it possible to be original in the development or application of ideas to solve novel or multidisciplinary engineering problems, after analysing and understanding the specifications put forward.[24]  Identify the effects of social, behavioral, legal, psychological, management, cognitive, and economic theories, methods, and models applicable to health informatics from multiple levels including individual, social group, and society.[21] |
| 2 | Applies concepts of nursing theory and research to the design of health information applications and systems.[16]  Critical awareness of the wider multidisciplinary context of engineering and of knowledge issues at the interface between different fields.[22]  Discusses computer fundamentals (hardware, software, networks, data communications).[16]  Differentiates significant highlights in the evolution of computer technology.[16]  Projects health care computing trends in nursing.[16]  Ability to analyse new and complex engineering products, processes and systems within broader or multidisciplinary contexts; to select and apply the most appropriate and relevant methods from established analytical, computational and experimental methods or new and innovative methods; to critically interpret the outcomes of such analyses.[22]  In-depth knowledge and understanding of mathematics, computing and sciences underlying their engineering specialisation, at a level necessary to achieve the other programme outcomes.[22]  Know the different fields of real application of artificial intelligence and be able to develop simple practical applications in some of them.[23]  Ability to apply acquired knowledge and solve problems in new or unfamiliar environments within broader, multidisciplinary contexts, being able to integrate this knowledge.[23]  To understand the fundamentals, history, principles and applications of intelligent systems.[23]  Be aware of new trends in robotic systems, especially in industrial robots, humanoid robots, bio-inspired robots, nano- and micro-robotics, social robotics, telerobotics, assistive robots and know the fields of application in which they are effective.[23]  Know the historical evolution of robots, classification, types, structure and morphology of robots. Identify and know the functionality of the components of a robot.[24]  Know how to solve engineering problems by applying knowledge of mathematics, physics, chemistry, computer science, design, mechanical, electrical, electronic and automatic systems to establish viable solutions in the field of the degree.[24]  Identify the effects of social, behavioural, legal, psychological, management, cognitive, and economic theories, methods, and models applicable to health informatics from multiple levels including individual, social group, and society.[21]  Identify the applicable information science and technology concepts, methods, and tools, which may be dependent upon the application area of the training program, to solve health informatics problems. These include the concepts, methods, and tools related to managing data, information, and knowledge, the basic information and computer science terms and concepts, the principles of information security, as well as the methods of assessing users’ information needs.[21]  Describe the history, goals, methods (including data and information used and produced), and current challenges of the major health science fields. These include biology, genomics, clinical and translational science, healthcare delivery, personal health, and population health.[21] |
| 3 | Determines the impact of information management technologies on therapeutic outcomes and quality of care.[16]  Discusses current applications available to support clinical care.[16]  Applies advanced methodological and statistical techniques to the design and evaluation of computerized clinical information systems.[16]  Comprehensive understanding of applicable techniques and methods of analysis, design and investigation and of their limitations.[22]  Ability to gather and interpret relevant data and handle complexity within their field of study, to inform judgements that include reflection on relevant social and ethical issues.[22]  Ability to analyse complex engineering products, processes and systems in their field of study; to select and apply relevant methods from established analytical, computational and experimental methods; to correctly interpret the outcomes of such analyses.[22]  Know, understand and know how to apply methodologies of analysis and validation of business opportunities in the field of robotics.[24]  Ability to investigate in a creative way the application of new and emerging technologies at the forefront of their engineering specialisation.[22]  Be aware of new trends in robotic systems, especially in industrial robots, humanoid robots, bio-inspired robots, nano- and micro-robotics, social robotics, telerobotics, assistive robots and know the fields of application in which they are effective.[23]  Identify the applicable information science and technology concepts, methods, and tools, which may be dependent upon the application area of the training program, to solve health informatics problems. These include the concepts, methods, and tools related to managing data, information, and knowledge, the basic information and computer science terms and concepts, the principles of information security, as well as the methods of assessing users’ information needs.[21]  Describe the history, goals, methods (including data and information used and produced), and current challenges of the major health science fields. These include biology, genomics, clinical and translational science, healthcare delivery, personal health, and population health.[21]  Demonstrate awareness of the value of information literacy and lifelong learning, maintenance of skills, and professional excellence.[21] |
| 4 | Interprets current legislation, research, and economics affecting computerized information management in health care.[16]  Determines the social, legal, and ethical impacts of changing to computerized information management.[16]  Ability to consult and apply codes of practice and safety regulations.[22]  Ability to integrate knowledge and handle complexity, to formulate judgements with incomplete or limited information, that include reflecting on social and ethical responsibilities linked to the application of their knowledge and judgement to deliver sustainable solutions for society, the economy and environment.[22]  Know and use safety measures for industrial or service robotic environments where people are involved, taking into account the relevant technical standards in this respect and ethical considerations where relevant.[24]  Define and discuss ethical principles and the informatician’s responsibility to the profession, their employers, and ultimately to the stakeholders of the informatics solutions they create and maintain.[21]  Demonstrate professional practices that incorporate ethical principles and values of the discipline.[21] |
| 5 | Includes client needs in requirements development.[16]  Ability to develop and design complex products (devices, artefacts, etc.), processes and systems in their field of study to meet established requirements, that can include an awareness of non-technical – societal, health and safety, environmental, economic and industrial– considerations; to select and apply relevant design methodologies.[22]  Explain the possibilities and limitations of today's robots.[23]  Knowing the technological needs of society and industry, and being able to improve services and production processes by applying current robotics technology, through the choice, acquisition and implementation of robotic systems in different applications, both industrial and services.[24]  Demonstrate consideration of the advantages and limitations of using information science and technology to solve biomedical and health information problems as well as the needs of the different stakeholders and context.[21] |
| 6 | Develops implementation plans[16]  Applies principles of computing (e.g., reading an algorithm)[16]  Apply different techniques of knowledge representation and reasoning to problem solving.[23]  Design algorithms and code them with different programming techniques, especially in robotic systems.[24] |
| 7 | Analyzes the impacts of information management technologies on time allocation and tasks of care.[16]  Consults in the design or enhancements to integrated patient information, management, educational or research systems.[16]  Supports integration of a unified nursing language with the standardized language developed in collaboration with other health care disciplines.[16]  Conducts basic science research to support the theoretical development of the informatics specialty (e.g., decision-making, human-computer interaction, taxonomy development, etc.).[16]  Integrates nursing taxonomies, unified nomenclatures, and other data needed by nurses within database design.[16]  Ability to develop and design complex products (devices, artefacts, etc.), processes and systems in their field of study to meet established requirements, that can include an awareness of non-technical – societal, health and safety, environmental, economic and industrial– considerations; to select and apply relevant design methodologies.[22]  Apply different techniques of knowledge representation and reasoning to problem solving.[23]  Identify possible biomedical and health information science and technology methods and tools for solving a specific biomedical and health information problem.[21] |
| 8 | Consults in the design or enhancements to integrated patient information, management, educational or research systems.[16]  Designs unique technology or system alternatives for clinical care, education, administration and/or research.[16]  Ability to develop, to design new and complex products (devices, artefacts, etc.), processes and systems, with specifications incompletely defined and/or competing, that require integration of knowledge from different fields and non-technical – societal, health and safety, environmental, economic and industrial commercial – constraints; to select and apply the most appropriate and relevant design methodologies or to use creativity to develop new and original design methodologies.[22]  Possess and understand the knowledge that makes it possible to be original in the development or application of ideas to solve novel or multidisciplinary engineering problems, after analysing and understanding the specifications put forward.[24]  Design a solution to a biomedical or health information problem by applying computational and systems thinking, information science, and technology.[21] |
| 9 | Applies advanced analysis and design concepts to the system life cycle process.[16]  Applies principles of computer programming in order to communicate with software developers.[16]  Develops methods of data communication, hardware and software integration, and data transformation.[16]  Ability to design using knowledge and understanding at the forefront of their engineering specialisation.[22]  Comprehensive understanding of applicable materials, equipment and tools, engineering technologies and processes, and of their limitations.[22]  Explain the components of a robot (sensors, actuators, software, mechanical elements, etc.), how they function as individual elements, and how they function as a system.[23]  Ability to apply acquired knowledge and problem-solving skills in new or unfamiliar environments within broader, multidisciplinary contexts, being able to integrate this knowledge.[24]  Design algorithms and code them with different programming techniques, especially in robotic systems.[24]  Identify the theories, models, and tools from social, business, human factors, behavioral, and information sciences and technologies for designing, implementing, and evaluating health informatics solutions.[21] |
| 10 | Constructs guidelines for the purchase of software and hardware.[16]  Develops methods of data communication, hardware and software integration, and data transformation.[16]  Conocer y saber utilizar programas informáticos de diseño y visualización de esquemas de circuitos, estructuras y mecanismos.[24] |
| 11 | Determines new requirements according to the needs of the organization.[16]  Develops requirements for an integrated clinical, education, administration and/or research applications.[16]  Performs needs assessment for future requirements.[16]  Ability to develop, to design new and complex products (devices, artefacts, etc.), processes and systems, with specifications incompletely defined and/or competing, that require integration of knowledge from different fields and non-technical – societal, health and safety, environmental, economic and industrial commercial – constraints; to select and apply the most appropriate and relevant design methodologies or to use creativity to develop new and original design methodologies.[22]  Design a solution to a biomedical or health information problem by applying computational and systems thinking, information science, and technology.[21] |
| 12 | Interprets capabilities and limitations of hardware, interfaces and their relationship to the outcomes of health computing.[16]  Comprehensive understanding of applicable techniques and methods of analysis, design and investigation and of their limitations.[22]  Be able to obtain and analyse information on the characteristics of materials, circuits, machine elements, automatic control, sensors and computer systems, with the ultimate aim of achieving autonomous and flexible robotic applications.[24] |
| 13 | Uses software tools as appropriate during the systems life cycle.[16]  Develops methods of data communication, hardware and software integration, and data transformation.[16]  Analyzes user areas to determine procedural errors versus hardware and software problems.[16]  Practical skills, including the use of computer tools, for solving complex problems, realising complex engineering design, designing and conducting complex investigations.[22]  Have knowledge of the fundamental aspects of materials science and technology most suitable for the construction of robots of different types.[24]  Verify the correct functioning of a programme.[24]  Integrate and apply the theories, models, and tools from social, business, human factors, behavioral, and information sciences and technologies to design, implement, and evaluate health informatics solutions.[21] |
| 14 | Devises strategies to involve clinicians in the design, selection, implementation, and evaluation of applications and systems in health care.[16]  Critical awareness of economic, organisational and managerial issues (such as project management, risk and change management).[22]  Apply relationship‐building skills and the principles of interprofessional communication in a responsive and responsible manner that supports a team approach to solve complex health and health information problems.[21] |
| 15 | Determines project scope, objectives, and resources for each proposed application, system or enhancement.[16]  Applies principles and concepts of project management.[16]  Develops research designs to examine impacts of computer technology in nursing. [16]  Ability to manage complex technical or professional activities or projects that can require new strategic approaches, taking responsibility for decision making.[22]  Ability to engage in independent life-long learning.[22]  Ability to investigate in a creative way the application of new and emerging technologies at the forefront of their engineering specialisation.[22]  Employ leadership and followership methods, concepts, and tools to motivate others toward accomplishing a health informatics vision.[21]  Demonstrate awareness of the value of information literacy and lifelong learning, maintenance of skills, and professional excellence.[21] |
| [16] | Publishes findings from informatics-focused research to support the development of the specialty’s theoretical knowledge base.[16]  Disseminates new knowledge by informing colleagues of new developments and applications in nursing or healthcare informatics.[16]  Ability to conduct searches of literature, to consult and critically use databases and other sources of information, to carry out simulation in order to pursue detailed investigations and research of complex technical issues.[22]  Advanced laboratory/workshop skills and ability to design and conduct experimental investigations, critically evaluate data and draw conclusions.[22] |
| 17 | Develops policies, procedures and guidelines based on research.[16]  Develops policies and procedures related to information systems implementation, use, and maintenance.[16]  Knowing the technological needs of society and industry, and being able to improve services and production processes by applying current robotics technology, through the choice, acquisition and implementation of robotic systems in different applications, both industrial and services.[22] |
