# Supplementary material for: Competencies in the Robotics of Care for Nursing Robotics: A Scoping Review
Source: Healthcare (Basel). 2024 Mar 8;12(6):617. doi: 10.3390/healthcare12060617 (PMC10970049; doi:10.3390/healthcare12060617)
Supplement: Supplementary file 1 [file healthcare-12-00617-s001.zip › HC ROB Supplementary Tables S2.docx]

**Supplementary Tables S2.** Competencies and Programme Outcomes selected.

**2.1 Competency Themes (Strudwick) [20]:**

| **ID** | **Competency theme** |
| --- | --- |
| 1 | Knowledge / Standarization |
| 2 | Information Systems |
| 3 | Education |
| 4 | Research |
| 5 | Ethical/legal/regulatory |
| 6 | Privacy/security knowledge |
| 7 | Impact |
| 8 | Requeriments |
| 9 | Implementation |
| 10 | Analysis/Evaluation |
| 11 | Non-informatics-specific competencies |

**2.2 Competencies in nursing informatics (Staggers) [16]:**

| **Informatics Specialist** | | | |
| --- | --- | --- | --- |
| **Code** | **Definition** | **Area 1** | **Area 2** |
| IS01 | Develops or modifies spreadsheets used for complex problems | Computer Skills | Basic Desktop Software |
| IS02 | Writes macros, shortcuts for spreadsheets | Computer Skills | Basic Desktop Software |
| IS03 | Manages projects with project management software | Computer Skills | Project Management |
| IS04 | Determines data indicators used to monitor quality and effectiveness of nursing informatics practice | Computer Skills | Quality Improvement |
| IS05 | Collects data to monitor quality and effectiveness of nursing informatics practice | Computer Skills | Quality Improvement |
| IS06 | Determines aspects of nursing informatics practice important for quality monitoring | Computer Skills | Quality Improvement |
| IS07 | Has the ability to integrate different applications or programs | Computer Skills | Systems |
| IS08 | Uses utility programs for data recovery and system performance indices | Computer Skills | Systems |
| IS09 | Demonstrates fluency in informatics and nursing terminologies | Informatics Knowledge | Data |
| IS10 | Supports integration of a unified nursing language with the standardized language developed in collaboration with other health care disciplines | Informatics Knowledge | Data |
| IS11 | Recognizes the capacity for data aggregation and integration | Informatics Knowledge | Data |
| IS12 | Implements and evaluates application/system training programs for users and clients | Informatics Knowledge | Education |
| IS13 | Plans and develops application/system training programs for users, clients | Informatics Knowledge | Education |
| IS14 | Constructs guidelines for the purchase of software and hardware | Informatics Knowledge | Education |
| IS15 | Participates with practicing nurses, nurse administrators, and nurse researchers to define and develop new computer competencies | Informatics Knowledge | Education |
| IS16 | Teaches users/clients about effective and ethical uses of applications and systems | Informatics Knowledge | Education |
| IS17 | Serves as an informational resource person for applications/system | Informatics Knowledge | Education |
| IS18 | Determines the impact of computerized information management on managers and executive roles | Informatics Knowledge | Impact |
| IS19 | Interprets current legislation, research, and economics affecting computerized information management in health care | Informatics Knowledge | Impact |
| IS20 | Assesses current capabilities and limitations of technology (e.g., data transfer rates, chip capacity) | Informatics Knowledge | Impact |
| IS21 | Determines projected impacts to users and organizations when changing to computerized information management | Informatics Knowledge | Impact |
| IS22 | Determines the reasons for slow response time (e.g., heavy demands on computer system at time of shift change | Informatics Knowledge | Impact |
| IS23 | Discusses new careers available to informaticists | Informatics Knowledge | Impact |
| IS24 | Determines the social, legal, and ethical impacts of changing to computerized information management | Informatics Knowledge | Impact |
| IS25 | Discusses the interdependencies computerized information management creates (e.g., changes when MD enters own orders) | Informatics Knowledge | Impact |
| IS26 | Determines the limitations, reliability of computerized patient monitoring systems | Informatics Knowledge | Impact |
| IS27 | Applies strategies for change management to produce satisfied and productive users | Informatics Knowledge | Impact |
| IS28 | Determines the impact of information management technologies on therapeutic outcomes and quality of care | Informatics Knowledge | Impact |
| IS29 | Discusses the computer's effect on cost of health care | Informatics Knowledge | Impact |
| IS30 | Interprets the benefits and risks of computerized information management | Informatics Knowledge | Impact |
| IS31 | Interprets research findings about the impact of computerized information management on clinical practice, educational, administration &/or research | Informatics Knowledge | Impact |
| IS32 | Analyzes the impacts of information management technologies on time allocation and tasks of care | Informatics Knowledge | Impact |
| IS33 | Interprets the impact of computerized information management on nursing education | Informatics Knowledge | Impact |
| IS34 | Interprets copyright issues in computing | Informatics Knowledge | Privacy/security |
| IS35 | Discusses features, capabilities and scope of user passwords | Informatics Knowledge | Privacy/security |
| IS36 | Devises strategies to protect the confidentiality of computerized information | Informatics Knowledge | Privacy/security |
| IS37 | Differentiates issues surrounding confidentiality in computerized information management | Informatics Knowledge | Privacy/security |
| IS38 | Incorporates relevant law and regulations into informatics practice | Informatics Knowledge | Regulations |
| IS39 | Explains various input and output devices | Informatics Knowledge | Systems |
| IS40 | Applies theories that influence computerization in health care | Informatics Knowledge | Systems |
| IS41 | Discusses computer fundamentals (hardware, software, networks, data communications) | Informatics Knowledge | Systems |
| IS42 | Projects health care computing trends in nursing | Informatics Knowledge | Systems |
| IS43 | Evaluates applications/systems available in health care | Informatics Knowledge | Systems |
| IS44 | Differentiates significant highlights in the evolution of computer technology | Informatics Knowledge | Systems |
| IS45 | Interprets capabilities and limitations of hardware, interfaces and their relationship to the outcomes of health computing | Informatics Knowledge | Systems |
| IS46 | Demonstrates extensive knowledge of the applications/systems currently in use | Informatics Knowledge | Systems |
| IS47 | Constructs resources to support users | Informatics Knowledge | Systems |
| IS48 | Discusses general knowledge of computer theory and terminology | Informatics Knowledge | Systems |
| IS49 | Recognizes viruses and other system risks | Informatics Knowledge | Systems |
| IS50 | Discusses broad knowledge of other available hardware and software | Informatics Knowledge | Systems |
| IS51 | Devises strategies to involve clinicians in the design, selection, implementation, and evaluation of applications and systems in health care | Informatics Knowledge | Systems |
| IS52 | Discusses current applications available to support clinical care | Informatics Knowledge | Systems |
| IS53 | Discusses concepts of telemedicine and Internet and their relationship to nursing | Informatics Knowledge | Systems |
| IS54 | Discusses bedside terminals and associated issues such as use in sterile environments | Informatics Knowledge | Systems |
| IS55 | Conducts site visits of health information systems in actual use | Informatics Knowledge | Systems |
| IS56 | Recommends who generates, owns, and uses nursing and other data | Informatics Knowledge | Systems |
| IS57 | Interprets the current and projected future state of physiological monitoring | Informatics Knowledge | Systems |
| IS58 | Analyzes the health and safety aspects of the work station and its location | Informatics Knowledge | Usability |
| IS59 | Applies human factors and ergonomics to the design of the computer screen, location and design of devices, and design of software | Informatics Knowledge | Usability |
| IS60 | Develops and implements work plans during application development and implementation | Informatics Skills | Analysis |
| IS61 | Constructs data elements appropriate to a given practice context | Informatics Skills | Analysis |
| IS62 | Applies principles and techniques of systems analysis | Informatics Skills | Analysis |
| IS63 | Discusses functional areas and how their information flow interacts with an area being evaluated | Informatics Skills | Analysis |
| IS64 | Analyzes current computerized information and recommends enhancements | Informatics Skills | Analysis |
| IS65 | Analyzes business practices to determine need for reengineering the information flow | Informatics Skills | Analysis |
| IS66 | Applies principles of computing (e.g., reading an algorithm) | Informatics Skills | Analysis |
| IS67 | Analyzes user areas to determine procedural errors versus hardware and software problems | Informatics Skills | Analysis |
| IS68 | Interprets information flow within the organization | Informatics Skills | Analysis |
| IS69 | Modifies existing applications, devices to meet changing requirements | Informatics Skills | Analysis |
| IS70 | Conducts feasibility assessments throughout the information systems life cycle | Informatics Skills | Analysis |
| IS71 | Prepares process flow charts to describe current and proposed information flows for all aspects of clinical systems | Informatics Skills | Analysis |
| IS72 | Analyzes organizations to determine policies affecting information flow | Informatics Skills | Analysis |
| IS73 | Determines problems and impediments in installing computerized information management | Informatics Skills | Analysis |
| IS74 | Constructs data structures and maintains data sets | Informatics Skills | Data / Data Structures |
| IS75 | Applies data structure concepts in designing a database system | Informatics Skills | Data / Data Structures |
| IS76 | Determines relationships among tables in databases and performs tasks such as database normalization | Informatics Skills | Data / Data Structures |
| IS77 | Integrates nursing taxonomies, unified nomenclatures, and other data needed by nurses within database design | Informatics Skills | Data / Data Structures |
| IS78 | Develops procedures to establish and maintain the validity and integrity of data and databases | Informatics Skills | Data / Data Structures |
| IS79 | Modifies available software programs to support data aggregation and analyses | Informatics Skills | Data / Data Structures |
| IS80 | Alters a defined data structure to interface with another data structure | Informatics Skills | Data / Data Structures |
| IS81 | Develops screen layouts, report formats and custom views of clinical data working directly with clinical departments and individual users | Informatics Skills | Design, development |
| IS82 | Consults in the design or enhancements to integrated patient information, management, educational or research systems | Informatics Skills | Design, development |
| IS83 | Participates in the development of new methods or in making modifications to improve the efficiency and/or effectiveness of data storage and its communication | Informatics Skills | Design, development |
| IS84 | Coordinates the development of integrated computer-based patient record technologies | Informatics Skills | Design, development |
| IS85 | Maintains database (e.g., adding, deleting fields, structuring input for others, relational database) | Informatics Skills | Design, development |
| IS86 | Incorporates established data and database management standards into database design Participates in the development of new tools for management purposes | Informatics Skills | Design, development |
| IS87 | Develops methods of data communication, hardware and software integration, and data transformation | Informatics Skills | Design, development |
| IS88 | Develops database structures to support clinical care, education, administration or research | Informatics Skills | Design, development |
| IS89 | Applies concepts of nursing theory and research to the design of health information applications and systems | Informatics Skills | Design, development |
| IS90 | Develops databases to facilitate clinical care, education, administration or research | Informatics Skills | Design, development |
| IS91 | Develops new ways to interact with information technology and access data | Informatics Skills | Design, development |
| IS92 | Assists in the development of computer applications to meet clinical, education, administration and research requirements | Informatics Skills | Design, development |
| IS93 | Applies skills in the systems life cycle to support all computer-enabled patient care activities. | Informatics Skills | Design, development |
| IS94 | Evaluates existing technologies for cost-effectiveness | Informatics Skills | Evaluation |
| IS95 | Evaluates data storage capacities of the system in use | Informatics Skills | Evaluation |
| IS96 | Assures that information systems used in the organization comply with standards set forth by external licensing, accreditation & regulatory agencies | Informatics Skills | Evaluation |
| IS97 | Evaluates hardware, software, and vendor support | Informatics Skills | Evaluation |
| IS98 | Participates on interdisciplinary teams that evaluate nursing informatics practice or health informatics services | Informatics Skills | Evaluation |
| IS99 | Analyzes the system in use | Informatics Skills | Evaluation |
| IS100 | Develops strategies to obtain funding for information systems | Informatics Skills | Fiscal Management |
| IS101 | Uses strategies to optimize application use after implementation (benefits realization) | Informatics Skills | Fiscal Management |
| IS102 | Participates in budget activities for the procurement and maintenance of the system | Informatics Skills | Fiscal Management |
| IS103 | Determines the cost-benefit of computer technology used in practice, education, administration and/or research | Informatics Skills | Fiscal Management |
| IS104 | Leads or participates in user groups during all phases of the systems life cycle | Informatics Skills | Implementation |
| IS105 | Devises strategies for installing applications/systems | Informatics Skills | Implementation |
| IS106 | Develops implementation plans | Informatics Skills | Implementation |
| IS107 | Distinguishes implementation phases (i.e., pre-implementation, implementation, post- implementation) | Informatics Skills | Implementation |
| IS108 | Applies installation tools during implementation | Informatics Skills | Implementation |
| IS109 | Develops information management plans and/or work plans to support the systems life cycle | Informatics Skills | Implementation |
| IS110 | Applies appropriate implementation strategies | Informatics Skills | Implementation |
| IS111 | Manages the installation process | Informatics Skills | Implementation |
| IS112 | Recognizes opportunities for applying information management technologies to clinical practice, education, administration and/or research situations | Informatics Skills | Implementation |
| IS113 | Devises strategies to encourage interdisciplinary use of computerized information management | Informatics Skills | Implementation |
| IS114 | Manages terms and conditions of a contract with an information systems vendor | Informatics Skills | Management |
| IS115 | Develops a plan for limited resources (e.g., costs, staffing, equipment) | Informatics Skills | Management |
| IS116 | Determines project scope, objectives, and resources for each proposed application, system or enhancement | Informatics Skills | Management |
| IS117 | Develops system testing, implementation, conversion, and backup plans | Informatics Skills | Management |
| IS118 | Develops a strategic or long-range plan for the management of applications and systems | Informatics Skills | Management |
| IS119 | Develops policies, procedures and guidelines based on research | Informatics Skills | Management |
| IS120 | Develops policies and procedures related to information systems implementation, use, and maintenance | Informatics Skills | Management |
| IS121 | Escalates client issues and problems to the next available level of management when appropriate | Informatics Skills | Management |
| IS122 | Communicates progress of project to appropriate personnel | Informatics Skills | Management |
| IS123 | Applies principles and concepts of project management | Informatics Skills | Management |
| IS124 | Functions as a project manager | Informatics Skills | Management |
| IS125 | Develops policies related to privacy, confidentiality and security of patient and client data | Informatics Skills | Privacy/security |
| IS126 | Recommends procedures for achieving data integrity and security | Informatics Skills | Privacy/security |
| IS127 | Analyzes the capability of information technology to support programs of data integrity and security | Informatics Skills | Privacy/security |
| IS128 | Determines the characteristics of a good computer program | Informatics Skills | Programming |
| IS129 | Applies principles of computer programming in order to communicate with software developers | Informatics Skills | Programming |
| IS130 | Differentiates between machine and high-level programming languages | Informatics Skills | Programming |
| IS131 | Determines priorities for new requirements within budget constraints | Informatics Skills | Requeriments |
| IS132 | Modifies information technologies to meet changing data requirements/needs | Informatics Skills | Requeriments |
| IS133 | Determines new requirements according to the needs of the organization | Informatics Skills | Requeriments |
| IS134 | Demonstrates skills in the systems life cycle to support policies, procedures and knowledge bases in organizations | Informatics Skills | Requeriments |
| IS135 | Includes client needs in requirements development | Informatics Skills | Requeriments |
| IS136 | Develops requirements for an integrated clinical, education, administration and/or research applications | Informatics Skills | Requeriments |
| IS137 | Communicates informatics' needs to a systems analyst | Informatics Skills | Requeriments |
| IS138 | Performs needs assessment for future requirements | Informatics Skills | Requeriments |
| IS139 | Influences change to improve the impact of informatics on the system of care | Informatics Skills | Role |
| IS140 | Designs strategies to manage the impact of change to information systems implementation. | Informatics Skills | Role |
| IS141 | Consults about informatics with clinical, managerial, educational, and/or research entities | Informatics Skills | Role |
| IS142 | Develops collegial relationships with information system technical support personnel | Informatics Skills | Role |
| IS143 | Serves as a liaison among agency departments and vendors | Informatics Skills | Role |
| IS144 | Collaborates with nursing personnel and interdisciplinary teams to accomplish information management work | Informatics Skills | Role |
| IS145 | Promotes understanding and effective use of information technology | Informatics Skills | Role |
| IS146 | Makes formal presentations of project findings, recommendations, and specifications to user department managers, supervisors, and/or administrators | Informatics Skills | Role |
| IS147 | Recommends changes in health informatics practice based upon evaluation data from nursing informatics (e.g., a validated severity of illness instrument) | Informatics Skills | Role |
| IS148 | Recommends policies and procedures to improve the quality of nursing informatics practice | Informatics Skills | Role |
| IS149 | Implements activities to enhance the quality of nursing informatics practice | Informatics Skills | Role |
| IS150 | Develops recommendations to improve nursing informatics practice or outcomes | Informatics Skills | Role |
| IS151 | Acts as a liaison to support communication among providers, patient, and technical communities | Informatics Skills | Role |
| IS152 | Uses software tools as appropriate during the systems life cycle | Informatics Skills | Role |
| IS153 | Provides backup support to installation personnel as required | Informatics Skills | Role |
| IS154 | Applies knowledge of patient care processes to systems and their life cycle | Informatics Skills | Role |
| IS155 | Maintains a system perspective that encompasses the entire organization | Informatics Skills | Role |
| IS156 | Integrates knowledge from other informatics disciplines with nursing to improve patient care, administration, education and/or research | Informatics Skills | Role |
| IS157 | Participates in top level decisions and policy design which impact clinical information management | Informatics Skills | Role |
| IS158 | Conducts research to examine impacts of computer technology in nursing | Informatics Skills | Role |
| IS159 | Conducts research to determine application needs in clinical care, education, administration and research | Informatics Skills | Role |
| IS160 | Conducts research in informatics | Informatics Skills | Role |
| IS161 | Disseminates new knowledge by informing colleagues of new developments and applications in nursing or healthcare informatics | Informatics Skills | Role |
| IS162 | Contributes to informatics education of students, peers and colleagues | Informatics Skills | Role |
| IS163 | Assists in the resolution of basic software problems | Informatics Skills | Systems Maintenance |
| IS164 | Performs complex trouble-shooting in applications | Informatics Skills | Systems Maintenance |
| IS165 | Recommends solutions to application-specific problems | Informatics Skills | Systems Maintenance |
| IS166 | Maintains the data dictionary and other technical support elements | Informatics Skills | Systems Maintenance |
| IS167 | Designs evaluation criteria and strategies for selecting applications and systems | Informatics Skills | System Selection |
| IS168 | Applies ergonomics principles in the selection and use of information management technologies | Informatics Skills | System Selection |
| IS169 | Participates with others in selecting applications or systems (e.g., users, vendors, system designers) | Informatics Skills | System Selection |
| IS170 | Develops procedures and scenarios for acceptance testing, conversions, and interface testing | Informatics Skills | Skills-Testing |
| IS171 | Conducts tests of information management applications, systems | Informatics Skills | Skills-Testing |
| IS172 | Produces short-term and long-term training plans | Informatics Skills | Skills-Training |
| IS173 | Produces training materials and operating manuals tailored to the organization | Informatics Skills | Skills-Training |
| IS174 | Delivers user training programs | Informatics Skills | Skills-Training |
| IS175 | Evaluates user training programs | Informatics Skills | Skills-Training |
| **Informatics Innovator** | | | |
| **Code** | **Definition** | **Area 1** | **Area 2** |
| II01 | Develops models for simulation purposes | Computer Skills | Simulation |
| II02 | Evaluates informatics competencies required for specific role functions for the practicing nurse, nurse administrator and others | Informatics Knowledge | Education |
| II03 | Evaluates the changing role of educator when computerized information management is introduced | Informatics Knowledge | Impact |
| II04 | Designs innovative analytic techniques | Informatics Skills | Analysis |
| II05 | Designs unique technology or system alternatives for clinical care, education, administration and/or research | Informatics Skills | Design, development |
| II06 | Develops the conceptual model for a database | Informatics Skills | Design, development |
| II07 | Evaluates the performance and impact of information management technologies on organizational efficiency | Informatics Skills | Evaluation |
| II08 | Evaluates factors related to safety, effectiveness, cost and social impact when developing and implementing information management technologies | Informatics Skills | Evaluation |
| II09 | Based upon information management technologies evaluation data, recommends and/or modifies clinical practice enhancements | Informatics Skills | Evaluation |
| II10 | Evaluates the performance and impact of information management technologies on clinical practice, education, administration &/or research | Informatics Skills | Evaluation |
| II11 | Develops a framework(s) for evaluating applications and system performance in clinical care, education, research, and/or administration | Informatics Skills | Evaluation |
| II12 | Develops strategies to obtain research funding | Informatics Skills | Fiscal Management |
| II13 | Designs innovative methods for project management | Informatics Skills | Management |
| II14 | Develops innovative and analytic techniques for scientific inquiry in nursing informatics | Research | Research |
| II15 | Develops new methods of organizing data to enhance research capacities | Research | Research |
| II16 | Develops research designs to examine impacts of computer technology in nursing. | Research | Research |
| II17 | Conducts basic science research to support the theoretical development of the informatics specialty (e.g., decision-making, human-computer interaction, taxonomy development, etc.) | Research | Research |
| II18 | Designs evaluation techniques to assess the quality of data and information in information systems (e.g., the validity of Internet-based patient educational content). | Research | Research |
| II19 | Applies advanced methodological and statistical techniques to the design and evaluation of computerized clinical information systems | Research | Research |
| II20 | Publishes findings from informatics-focused research to support the development of the specialty’s theoretical knowledge base | Research | Research |
| II21 | Sustains an informatics-focused program of research | Research | Research |
| II22 | Applies multivariate statistical concepts to the evaluation of complex data sets to forecast quality management trends | Research | Research |
| II23 | Develops psychometrically sound instruments for use in informatics-focused research. | Research | Research |
| II24 | Develops new framework(s) for use in informatics | Research | Research |
| II25 | Applies advanced analysis and design concepts to the system life cycle process | Practice | Practice |
| II26 | Integrates domain knowledge within computerized decision support systems | Practice | Practice |
| II27 | Analyzes complex issues (e.g., confidentiality, privacy, and data security) | Practice | Practice |
| II28 | Recommends policies based upon analytical findings | Practice | Practice |
| II29 | Designs and/or evaluates enterprise-wide strategies for managing the impact of information systems implementation | Practice | Practice |
| II30 | Designs the structure for complex data sets | Practice | Practice |
| II31 | Develops new methods of organizing data to enhance research capabilities | Practice | Practice |
| II32 | Develops innovative methods of data communication, hardware and software integration, and data transformation | Practice | Practice |
| II33 | Designs unique system alternatives for clinical care, education, administration or research | Practice | Practice |
| II34 | Exerts leadership of interdisciplinary teams to provide strategic IS direction | Practice | Practice |
| II35 | Influences top-level decisions and policy design which impact clinical information management | Practice | Practice |
| II36 | Applies sophisticated educational design and research evaluation concepts to the use of innovative computer-based education techniques (e.g., distance education) | Education | Education |
| II37 | Develops theoretically-based curricular models for nursing informatics | Education | Education |

**2.3 Programme Outcomes EUR-ACE^®^ Framework [22]:**

| **Programme Outcomes describe the knowledge, understanding, skills and abilities which an accredited engineering degree programme must enable a graduate to demonstrate.** | | | | |
| --- | --- | --- | --- | --- |
| **IDBD** | **Bachelor Degree Programs** | **IDMD** | **Master Degree Programmes** | **Cathegories** |
| **B1** | Knowledge and understanding of the mathematics, computing and other basic sciences underlying their engineering specialisation, at a level necessary to achieve the other programme outcomes; | **M1** | In-depth knowledge and understanding of mathematics, computing and sciences underlying their engineering specialisation, at a level necessary to achieve the other programme outcomes; | Knowledge and understanding; |
| **B2** | Knowledge and understanding of engineering fundamentals underlying their specialisation, at a level necessary to achieve the other programme outcomes, including some awareness at their forefront; | **M2** | In-depth knowledge and understanding of engineering disciplines underlying their specialisation, at a level necessary to achieve the other programme outcomes; | Knowledge and understanding; |
| **B3** | Awareness of the wider multidisciplinary context of engineering. | **M3** | Critical awareness of the forefront of their specialisation; | Knowledge and understanding; |
|  |  | **M4** | Critical awareness of the wider multidisciplinary context of engineering and of knowledge issues at the interface between different fields. | Knowledge and understanding; |
| **B5** | Ability to analyse complex engineering products, processes and systems in their field of study; to select and apply relevant methods from established analytical, computational and experimental methods; to correctly interpret the outcomes of such analyses; | **M5** | Ability to analyse new and complex engineering products, processes and systems within broader or multidisciplinary contexts; to select and apply the most appropriate and relevant methods from established analytical, computational and experimental methods or new and innovative methods; to critically interpret the outcomes of such analyses; | Engineering Analysis |
| **B6** | Ability to identify, formulate and solve engineering problems in their field of study; to select and apply relevant methods from established analytical, computational and experimental methods; to recognise the importance of non-technical –societal, health and safety, environmental, economic and industrial – constraints. | **M6** | Ability to identify, formulate and solve unfamiliar complex engineering problems that are incompletely defined, have competing specifications, may involve considerations from outside their field of study and non-technical – societal, health and safety, environmental, economic and industrial – constraints; to select and apply the most appropriate and relevant methods from established analytical, computational and experimental methods or new and innovative methods in problem solving; | Engineering Analysis |
|  |  | **M7** | Ability to conceptualise engineering products, processes and systems; | Engineering Analysis |
|  |  | **M8** | Ability to identify, formulate and solve complex problems in new and emerging areas of their specialisation. | Engineering Analysis |
| **B9** | Ability to develop and design complex products (devices, artefacts, etc.), processes and systems in their field of study to meet established requirements, that can include an awareness of non-technical – societal, health and safety, environmental, economic and industrial– considerations; to select and apply relevant design methodologies; | **M9** | Ability to develop, to design new and complex products (devices, artefacts, etc.), processes and systems, with specifications incompletely defined and/or competing, that require integration of knowledge from different fields and non-technical – societal, health and safety, environmental, economic and industrial commercial – constraints; to select and apply the most appropriate and relevant design methodologies or to use creativity to develop new and original design methodologies. | Engineering Design |
| **B10** | Ability to design using an awareness of the forefront of their engineering specialisation. | **M10** | Ability to design using knowledge and understanding at the forefront of their engineering specialisation. | Engineering Design |
| **B11** | Ability to conduct searches of literature, to consult and to critically use scientific databases and other appropriate sources of information, to carry out simulation and analysis in order to pursue detailed investigations and research of technical issues in their field of study; | **M11** | Ability to conduct searches of literature, to consult and critically use databases and other sources of information, to carry out simulation in order to pursue detailed investigations and research of complex technical issues; | Investigations |
|  |  | **M12** | Ability to identify, locate and obtain required data; | Investigations |
| **B13** | Ability to consult and apply codes of practice and safety regulations in their field of study; | **M13** | Ability to consult and apply codes of practice and safety regulations; | Investigations |
| **B14** | Laboratory/workshop skills and ability to design and conduct experimental investigations, interpret data and draw conclusions in their field of study. | **M14** | Advanced laboratory/workshop skills and ability to design and conduct experimental investigations, critically evaluate data and draw conclusions; | Investigations |
|  |  | **M15** | Ability to investigate in a creative way the application of new and emerging technologies at the forefront of their engineering specialisation. | Investigations |
| **B16** | Understanding of applicable techniques and methods of analysis, design and investigation and of their limitations in their field of study; | **M16** | Comprehensive understanding of applicable techniques and methods of analysis, design and investigation and of their limitations; | Engineering Practice |
| **B17** | Practical skills for solving complex problems, realising complex engineering designs and conducting investigations in their field of study; | **M17** | Practical skills, including the use of computer tools, for solving complex problems, realising complex engineering design, designing and conducting complex investigations; | Engineering Practice |
| **B18** | Understanding of applicable materials, equipment and tools, engineering technologies and processes, and of their limitations in their field of study; | **M18** | Comprehensive understanding of applicable materials, equipment and tools, engineering technologies and processes, and of their limitations; | Engineering Practice |
| **B19** | Ability to apply norms of engineering practice in their field of study; | **M19** | Ability to apply norms of engineering practice; | Engineering Practice |
| **B20** | Awareness of non-technical -societal, health and safety, environmental, economic and industrial – implications of engineering practice; | **M20** | Knowledge and understanding of the non-technical – societal, health and safety, environmental, economic and industrial – implications of engineering practice; | Engineering Practice |
| **B21** | Awareness of economic, organisational and managerial issues (such as project management, risk and change management) in the industrial and business context. | **M21** | Critical awareness of economic, organisational and managerial issues (such as project management, risk and change management) | Engineering Practice |
| **B22** | Ability to gather and interpret relevant data and handle complexity within their field of study, to inform judgements that include reflection on relevant social and ethical issues; | **M22** | Ability to integrate knowledge and handle complexity, to formulate judgements with incomplete or limited information, that include reflecting on social and ethical responsibilities linked to the application of their knowledge and judgement to deliver sustainable solutions for society, the economy and environment; | Making Judgements Communication and Team-working |
| **B23** | Ability to manage complex technical or professional activities or projects in their field of study, taking responsibility for decision making. | **M23** | Ability to manage complex technical or professional activities or projects that can require new strategic approaches, taking responsibility for decision making. | Making Judgements Communication and Team-working |
| **B24** | Ability to recognise the need for and to engage in independent life-long learning;ability to follow developments in science and technology. | **M24** | Ability to engage in independent life-long learning; | Lifelong Learning |
|  |  | **M25** | Ability to undertake further study autonomously. | Lifelong Learning |

**2.4 Programme Outcomes of White Book of Robotics in Spain and Robotics Engineering Graduate Programme [23,24]:**

| **Name of the degree** | **Subject / General or Specific competence** | ID | **Learning outcomes extracted from the Robotics White Paper (published in Spain in 2011) and the University of Alicante Robotics Engineering Graduate Study Plan (24 May 2022).** |
| --- | --- | --- | --- |
| **Industrial Engineering [32]**  Degree in Electronic and Automatic Engineering at the University of Zaragoza. | Industrial robotics subject | R1 | In-depth knowledge of the drive, sensory and control subsystems of an industrial robot. |
|  |  | R2 | To know the technical foundations for the design of the control and programming system of an industrial robot. |
|  |  | R3 | Acquire skills to model and programme an industrial robot. |
|  |  | R4 | Assess the convenience and viability of robotising production processes, taking into account economic, quality and safety aspects. |
|  |  | R5 | Knowing how to design a robotic cell, selecting the robot and integrating it with other elements of the production process, and designing the robotic application, using the programming language supplied with the robot. |
|  | Automation and robotics | R6 | To know and apply the basic techniques of mobile robotics. |
|  |  | R7 | Know and apply basic computer vision techniques. |
|  |  | R8 | Design and analyse discrete system control systems. |
|  |  | R9 | Know and apply real-time system development techniques. |
|  |  | R10 | Know how to use simulation as a technique for the analysis of both continuous and discrete systems. |
|  |  | R11 | Have a practical knowledge of the above aspects. |
| **Computer Engineering [32]**  Degree in Computer Engineering, University of Malaga. | Robotics encompassed in computing | R12 | Explain the possibilities and limitations of current robots. |
|  |  | R13 | Explain the components of a robot (sensors, actuators, software, mechanical elements, etc.), their functioning as individual elements, and their functioning as a system. |
|  |  | R14 | Explain and apply the basic methods of sensory data processing to capture information from the environment. |
|  |  | R15 | Explain and apply basic movement planning algorithms. |
|  |  | R16 | Program a robot to perform basic operations in not fully controlled environments. |
|  | Artificial Intelligence | R17 | To understand the fundamentals, history, principles and applications of intelligent systems. |
|  |  | R18 | Apply search techniques to solve problems and games with opponents. |
|  |  | R19 | Understand basic planning techniques and their practical application. |
|  |  | R20 | Apply different techniques of knowledge representation and reasoning to solve problems. |
|  |  | R21 | To understand the design principles and architectures of multi-agent cooperative systems. |
|  |  | R22 | Analyse which problems can be tackled using machine learning techniques, and apply them to simple cases. |
|  |  | R23 | Know the different fields of real application of artificial intelligence and be able to develop simple practical applications in some of them. |
|  | Learning | R24 | Know the basic machine learning techniques and explain their differences. |
|  |  | R25 | Determine which learning technique is most appropriate for a given problem. |
|  |  | R26 | Implement simple algorithms for supervised learning, reinforcement learning, and unsupervised learning on real problems. |
|  |  | R27 | Characterise the state of the art in machine learning, achievements and limitations. |
| **Degree in Robotics Engineering [33]** University of Alicante | General competences | R28 | Know how to solve engineering problems by applying knowledge of mathematics, physics, chemistry, computer science, design, mechanical, electrical, electronic and automatic systems to establish viable solutions in the field of the degree. |
|  |  | R29 | Ability to use computer tools for modelling, simulation and design of engineering applications. |
|  |  | R30 | Possess and understand the knowledge that makes it possible to be original in the development or application of ideas to solve novel or multidisciplinary engineering problems, after analysing and understanding the specifications put forward. |
|  |  | R31 | Knowing the technological needs of society and industry, and being able to improve services and production processes by applying current robotics technology, through the choice, acquisition and implementation of robotic systems in different applications, both industrial and services. |
|  |  | R32 | To be able to obtain and analyse information on the characteristics of materials, circuits, machine elements, automatic control, sensors and computer systems, with the ultimate aim of achieving autonomous and flexible robotic applications. |
|  |  | R33 | Conceiving, calculating, designing and implementing algorithms, equipment or installations in the field of robotics, for industrial or service applications, taking into account aspects of quality, safety, environmental criteria, rational and efficient use of resources. |
|  |  | R34 | Knowing how to apply new robotics technologies to different business sectors, especially industrial and service sectors, in order to improve their competitiveness. |
|  | Specific competences | R35 | To develop the student's ability to apply, both from an analytical and numerical point of view, knowledge of: Linear Algebra, Differential and Integral Calculus, Differential and Partial Derivative Equations as well as Complex Variables, to different mathematical problems that arise in robotic systems. |
|  |  | R36 | Understand and know how to apply to engineering problems the physical foundations on which robotics engineering is based: statics, kinematics, dynamics, mechanics, thermodynamics, electromagnetism and electrical circuits. |
|  |  | R37 | To know the main aspects of the structure and chemical and functional properties of materials in order to be able to determine the most suitable materials for robotics applications. |
|  |  | R38 | Know and evaluate the structure and basic components of computers. |
|  |  | R39 | Knowing how to use and integrate operating systems and embedded systems, as well as their multitasking or inter-application communication features. |
|  |  | R40 | Interpret the operation of the source code of a program. |
|  |  | R41 | Define the types of data needed for the representation of information. |
|  |  | R42 | Designing algorithms and coding them with different programming techniques, especially in robotic systems. |
|  |  | R43 | Verify the correct functioning of a programme. |
|  |  | R44 | Have the capacity for spatial vision and knowledge of graphic representation techniques, enabling the design and interpretation of plans of mechanical systems and electrical and electronic circuits. |
|  |  | R45 | Know and learn how to use computer programmes for the design and visualisation of circuit diagrams, structures and mechanisms. |
|  |  | R46 | Know the historical evolution of robots, classification, types, structure and morphology of robots. Identify and know the functionality of the components of a robot. |
|  |  | R47 | Understand the principles of structures, machines, mechanisms, joints and motion transmission systems, and know how to apply them in robotic systems engineering. |
|  |  | R48 | Knowing how hydraulic and pneumatic systems for robotic drives work, and to know how to apply them in the resolution of robotics applications. |
|  |  | R49 | Have knowledge of the fundamental aspects of materials science and technology most suitable for the construction of robots of different types. |
|  |  | R50 | Knowledge of metallic alloys, non-metallic materials, new trends and their structures and morphologies. |
|  |  | R51 | Know how to apply the principles of material strength and elastic behaviour (deformation, traction, tension, bending, joints) and be able to determine the most suitable materials for their strength and durability for their application in robotics. |
|  |  | R52 | Know the principles of circuit theory and the fundamentals of electrical engineering and electronics (analogue, digital and power), and be able to analyse existing circuits, or design new ones, for robotic or other auxiliary systems. |
|  |  | R53 | Know and understand the operation of electrical machines, especially AC and DC motors, and know how to apply them in the analysis and design of actuators in robotic systems. |
|  |  | R54 | Know the most appropriate mathematical tools and computer applications for the modelling and analysis of linear and non-linear systems, and be able to analyse their dynamic behaviour. |
|  |  | R55 | Being able to model and simulate aspects of kinematics, dynamics, structures and mechanisms in order to be able to design and analyse robotic systems. |
|  |  | R56 | Have the ability to deal with kinematics and dynamics problems associated with the design, construction and analysis of robots. |
|  |  | R57 | Knowing how to use and design algorithms to generate movement trajectories, with sufficient precision, to adequately position different types of robots. |
|  |  | R58 | To learn about different kinds of sensor devices used to capture information from the robot itself and its environment, as well as their operating principles. |
|  |  | R59 | Know how to apply methods and techniques to measure, process, merge and represent the information captured. |
|  |  | R60 | To know how the different types of actuators are controlled by means of amplifiers, servos, valves or variators, in order to know how to choose, use and programme the most suitable element. |
|  |  | R61 | Analyse and understand the configuration of an automatic control system in order to modify or upgrade it using techniques for designing, configuring and adjusting controllers. |
|  |  | R62 | To know how logic controllers or automata work and are programmed, and to know how to use them in the development of automatic robotic systems. |
|  |  | R63 | To know which are the most suitable energy sources for stationary or autonomous robots. |
|  |  | R64 | Understand the operation and characteristics of different autonomous energy sources, such as batteries, fuel cells or solar cells, and have the ability to select the appropriate one for each autonomous robotics application. |
|  |  | R65 | Be able to apply the techniques of kinematic and dynamic control, planning and programming of robots and other associated automation systems in different situations. |
|  |  | R66 | Know how to select a robot for implementation in an application taking into account existing specifications and standards. |
|  |  | R67 | Be aware of new trends in robotic systems, especially in industrial robots, humanoid robots, bio-inspired robots, nano- and micro-robotics, social robotics, telerobotics, assistive robots and know the fields of application in which they are effective. |
|  |  | R68 | Know and use safety measures for industrial or service robotic environments involving people, taking into account the relevant technical standards in this respect and ethical considerations where relevant. |
|  |  | R69 | To know the different means of locomotion applicable to robotics, their dynamic peculiarities and most suitable fields of application (wheels, caterpillars, legs, aerial and others). |
|  |  | R70 | Knowing the artificial intelligence techniques used in industrial and service robotics, knowing how to use them in fixed and mobile robotic applications. |
|  |  | R71 | To be able to apply pattern recognition and computational learning methods in the analysis of sensory data and for decision making in robotic systems. |
|  |  | R72 | Be able to apply techniques for interaction between robotic systems and humans. |
|  |  | R73 | To know the cognitive and learning systems that can be applied to robotics. |
|  |  | R74 | Know how to apply the principles of current network architectures, protocols and network technologies to communicate the elements of a robotic system with each other and with other computer equipment. |
|  |  | R75 | To know the characteristics and standards of communications for the industrial field, and to know how to choose the appropriate ones for robotics applications in special working environments. |
|  |  | R76 | Know and understand the techniques for detecting, recognising or tracking elements within a robot's environment, and know how to use or develop algorithms to implement these techniques. |
|  |  | R77 | Know how different types of navigation, localisation and mapping systems work, for robotic systems, and the fields of application in which they can be used (indoor, aerial, terrestrial, marine...) |
|  |  | R78 | Be able to set up cooperative and multi-robot robotic systems using appropriate techniques. |
|  |  | R79 | The ability to design and plan robotic systems and their implementation in industry and services. |
|  |  | R80 | Know, understand and know how to apply methodologies of analysis and validation of business opportunities in the field of robotics. |
| **Master's Degree in Computer Engineering [32].** Carlos III University of Madrid | Intelligent Systems Design | R81 | Ability to apply mathematical, statistical and artificial intelligence methods to model, design and develop applications, services, intelligent systems and knowledge-based systems. |
|  |  | R82 | Ability to plan, calculate and design intelligent systems. |
|  |  | R83 | Ability to apply acquired knowledge and solve problems in new or unfamiliar environments within broader, multidisciplinary contexts, being able to integrate this knowledge. |
